# Opioid Dependence Increases Complications and Costs Following Lumbar Spinal Fusion: Insights from a Nationwide Database

**DOI:** 10.3390/jcm14113929

**Published:** 2025-06-03

**Authors:** Assil Mahamid, Lior Laver, Liad Alfandari, Hamza Jabareen, Noa Martonovich, Amit Keren, Eyal Behrbalk

**Affiliations:** 1 Department of Orthopedic Surgery, Hillel Yaffe Medical Center, Hadera 3820302, Israelliadalfa@gmail.com (L.A.);; 2 Rappaport Faculty of Medicine, Technion University Hospital (Israel Institute of Technology), Haifa 3200003, Israel

**Keywords:** lumbar fusion, opioid dependence, National Inpatient Sample, big data

## Abstract

**Background:** Opioid dependence is prevalent among patients undergoing lumbar spinal fusion and has been linked to poor postoperative outcomes. However, its specific impact on surgical complications and hospital resource utilization remains unclear. This study evaluates the association between opioid dependence and postoperative complications, length of stay (LOS), and hospital charges in lumbar fusion patients. **Methods:** A retrospective analysis was conducted using the National Inpatient Sample (NIS) database from 2016 to 2021. Adult patients (aged > 18 years) who underwent lumbar fusion surgery were identified and categorized based on opioid dependence using ICD-10 codes. Propensity score weighting (PSW) was employed to balance baseline characteristics. Primary outcomes included inpatient mortality, LOS, hospital charges, and postoperative complications. Statistical analyses were performed using survey-weighted generalized linear models. **Results:** Among 597,455 lumbar fusion patients, 7715 (1.3%) had documented opioid dependence. After PSW, opioid-dependent patients had significantly increased odds of blood loss anemia (OR 1.79, *p* < 0.001), respiratory complications (OR 2.17, *p* < 0.001), surgical site infections (OR 3.94, *p* = 0.001), and cardiac complications (OR 1.53, *p* = 0.002). They also had higher hospital charges (mean difference USD 17,739.2, *p* < 0.001) and prolonged LOS (mean difference 0.83 days, *p* < 0.001). Differences in urinary tract infections, acute renal failure, and stroke were not statistically significant after PSW. **Conclusions:** Opioid dependence is associated with increased postoperative complications, longer hospital stays, and higher healthcare costs in lumbar fusion patients. These findings highlight the need for improved perioperative pain management and opioid stewardship strategies to optimize surgical outcomes.

## 1. Introduction

In 2017, an estimated 109,500 people worldwide lost their lives to opioid overdose. In the United States alone, opioid-related deaths surged to 81,806 in 2022, reflecting a rapidly intensifying public health crisis [1,2]. On a global scale, approximately 40.5 million individuals were dependent on opioids as of 2017, with around 2.4 million Americans living with active opioid use disorder by 2019 [1,3]. These staggering numbers highlight the heavy toll of opioid dependence and the pressing need for effective prevention strategies, treatment options, and public health policies to address the issue.

The link between opioid use and spine surgery is both significant and complex. More than half of patients scheduled for spine surgery report using opioids before their operation [4,5,6]. This preoperative opioid use is linked to poorer outcomes after surgery, including less improvement in pain and function, lower satisfaction rates, higher rates of complications within 90 days, and continued opioid use long after the procedure [4]. The Congress of Neurological Surgeons has also emphasized that patients using opioids prior to surgery tend to need more pain medication in the early recovery phase and often experience worse long-term functional recovery [7]. Patients with substantial preoperative opioid consumption also require higher opioid doses during the intraoperative and immediate postoperative periods, which complicates pain management, increases perioperative opioid demand, and may prolong hospital stays, further hindering recovery [4,8]. Opioid-sparing protocols for lumbar fusion surgery emphasize multimodal analgesia, regional anesthesia techniques, and local anesthetic infusions to reduce opioid use and enhance postoperative pain control [9,10,11,12,13].

Although concerns about opioid dependence in surgical populations are growing, there is still a lack of focused research on postoperative pain management specifically among opioid-dependent patients undergoing lumbar fusion (LF). Many earlier studies have grouped together various spinal procedures, including both lumbar and cervical surgeries, which makes it difficult to draw clear, direct comparisons [14]. One notable analysis using the National Inpatient Sample (NIS) from 2003 to 2014 included 7963 patients with opioid dependence [15]. Interestingly, our study—despite covering only six years—identified a nearly identical number of opioid-dependent patients (*n* = 7715), highlighting the increasing prevalence of opioid use among individuals undergoing LF surgery. Gaining a clearer understanding of how opioid dependence affects the outcomes and complications of LF is essential. While previous work has examined these issues, our study adds a more contemporary perspective by leveraging the most recent data available from the NIS, covering the years 2016 through 2021. This study will conduct a comparative analysis of lumbar fusion (LF) procedures among these populations, focusing on epidemiology, underlying etiology, patient demographics, comorbid conditions, postoperative complications, length of hospital stay, and associated hospitalization costs. By offering a comprehensive overview of outcome differences between these groups, the findings aim to support evidence-based clinical decision-making and enhance patient care strategies.

## 2. Methods

### 2.1. Data Source

This study utilized the National Inpatient Sample (NIS), a comprehensive database developed by the Agency for Healthcare Research and Quality (AHRQ) under the Healthcare Cost and Utilization Project (HCUP). The NIS is the largest publicly available all-payer inpatient healthcare database in the United States, systematically capturing approximately 20% of inpatient hospitalizations from HCUP-affiliated institutions. This dataset includes approximately 7 million unweighted admissions annually, enabling robust epidemiological analyses and national estimations when adjusted using the discharge sample weights provided by HCUP.

For this study, data from 1 January 2016 to 31 December 2021 were analyzed, representing the most recent period available at the time of investigation. Each data entry in the dataset, termed a “case”, represents a weighted discharge record corresponding to approximately five actual patient encounters in the national inpatient population. This weighting methodology ensures accurate extrapolation to national estimates, enhancing the generalizability and statistical reliability of the findings.

### 2.2. Cohort Definition and Selection Criteria

The National Inpatient Sample (NIS) database was queried for the period 2016–2021 to identify adult patients (aged > 18 years) who underwent LF. Surgical cases were categorized based on the presence or absence of opioid dependence on admission only, utilizing ICD-10 codes. A total of 119,491 cases of reverse shoulder replacement surgery were identified, corresponding to 597,455 weighted patient encounters. Given the de-identified nature of the NIS database, this study was exempt from institutional review board (IRB) approval, and the requirement for informed consent was waived.

### 2.3. Outcome Variables (End Points)

The primary objective of this study was to evaluate the impact of opioid dependence on postoperative outcomes and complications following LF. Key outcome measures included inpatient mortality, length of stay (LOS), hospital charges, and inpatient postoperative complications. The complications assessed in this study included venous thromboembolism, encompassing deep vein thrombosis (DVT) and pulmonary embolism (PE), as well as surgical site infections, cardiac complications such as myocardial infarction and cardiac arrest, respiratory complications including pneumonia and respiratory failure, and renal and urinary complications such as acute renal failure and urinary tract infection (UTI).

For analytical purposes, continuous variables such as LOS and hospital charges were dichotomized. Patients whose LOS exceeded the 75th percentile were classified as having prolonged LOS, while those whose hospital charges surpassed the 75th percentile were categorized as having high-end hospital charges. Total hospital charges were analyzed as reported in the NIS and were not converted to estimated costs using cost-to-charge ratios. Dollar values were not adjusted for inflation, as the primary objective was to compare relative differences between groups rather than assess absolute financial trends over time.

### 2.4. Statistical Analysis

Continuous variables were expressed as mean value ± standard deviation (mean ± SD) when the normal distribution was met; if not, median with interquartile range (IQR) was used. Continuous variables were analyzed using the two-sample *t*-test or nonparametric Wilcoxon test, while statistical analysis for categorical variables was performed using the Chi-square test or the Fisher exact test. Prior to primary analysis, missing data were handled using single multivariate imputation by the regression method. The percentage of missing data was low: age (0.17%), female sex (2.01%), total hospital charges (0.69%), with no missing data for other variables.

To minimize the impact of selection bias and potential confounding, we performed propensity score weighting using a generalized boosted model (GBM) for propensity score estimation. The propensity score model included demographics (age, sex), comorbidities (type 2 diabetes, hypertension, dyslipidemia, sleep apnea, chronic anemia, alcohol abuse, smoking status, chronic kidney disease, COPD), hospital characteristics (bed size, location/teaching status, region), and insurance type. The sampling weights from the original dataset were incorporated into the propensity score estimation. Balance between weighted groups was assessed using standardized mean differences (SMDs), with values < 0.1 indicating good balance. Survey-weighted generalized linear models were used to estimate the effects of opioid use on various postoperative outcomes, accounting for the propensity score weights. Survey-weighted generalized linear models (GLMs) were used to evaluate the association between opioid dependence and binary outcomes, including inpatient mortality, complications, prolonged length of stay (LOS), and high-end hospital charges. A log-binomial model was used when convergence allowed; otherwise, Poisson regression with a log link and robust standard errors was implemented to obtain relative risk estimates. For LOS and hospital charges, which were dichotomized based on the 75th percentile, the same GLM framework was used, treating these variables as binary outcomes. Exponentiated coefficients (adjusted relative risks) are reported in the main tables. Marginal estimates (average marginal effects) were not computed. All models incorporated NIS survey weights to account for the complex sampling design, ensuring nationally representative estimates and valid standard errors. All statistical analyses were conducted using R version 4.4.1; due to multiple comparisons, we applied a Bonferroni correction. A significance threshold of *p* < 0.005 was used, and 99.5% confidence intervals were reported.

### 2.5. Ethical Consideration

This study was granted exempt status by the institutional review board (IRB) due to the de-identified nature of the National Inpatient Sample (NIS) dataset, ensuring full compliance with ethical guidelines for human subject research. Artificial intelligence (AI) tools were utilized solely to enhance the linguistic clarity, grammatical accuracy, and stylistic refinement of the manuscript. These tools were not employed for data analysis, statistical interpretation, or content generation, thereby preserving the scientific integrity and methodological rigor of this study (Table 1).

## 3. Results

Among the 597,455 patients who underwent spinal surgery, 7715 (1.3%) were identified as opioid-dependent. As shown in Table 2, these patients tended to be younger, with a mean age of 58.2 years compared to 62.2 years among non-dependent patients (SD 12.09, *p* < 0.001), and were treated in different hospital environments. Specifically, opioid-dependent individuals were more often treated in large hospitals (63.6% vs. 47.2%, *p* < 0.001) and urban teaching facilities (80.2% vs. 73.4%, *p* < 0.001). Regional variation was also apparent: opioid-dependent patients were more commonly located in the West (29.6% vs. 18.7%) and Midwest (27.3% vs. 23.9%), but less frequently in the South (31.7% vs. 41.2%, *p* < 0.001 for regional distribution). Insurance coverage further highlighted disparities between groups. Although Medicare remained the most common payer for both, opioid-dependent patients had significantly higher Medicaid coverage (12.8% vs. 5.8%) and lower rates of private insurance (29.5% vs. 37.9%) (*p* < 0.001).

The comorbidity profile highlighted several notable differences between the groups. Opioid-dependent patients exhibited significantly higher rates of alcohol abuse (3.4% vs. 1.0%, *p* < 0.001) and chronic obstructive pulmonary disease (COPD) (12.8% vs. 7.4%, *p* < 0.001) (Table 3).

The propensity-weighted cohort analysis revealed important differences in complication rates and hospital resource utilization between opioid-dependent and non-dependent patients undergoing spinal surgery. Overall, the most common postoperative complication was blood loss, affecting 17.8% of patients, followed by cardiac complications (5.3%), respiratory issues (3.0%), acute renal failure (2.8%), and urinary tract infections (2.6%). Less frequent but clinically relevant complications included dural tears (0.9%), surgical site infections (0.2%), and stroke (0.2%). When stratified by opioid dependence, notable disparities emerged. Opioid-dependent patients experienced significantly higher rates of blood loss complications (20.5% vs. 15.0%) and respiratory complications (3.8% vs. 2.3%) compared to their non-dependent counterparts. These findings underscore a heightened vulnerability to postoperative morbidity in the opioid-dependent population.

Concerning hospital charges, approximately 35% of opioid-dependent patients incurred high hospital charges compared to 28% of their non-dependent counterparts, with a relative risk of 1.25 (99.5% CI, 1.10–1.42). Similarly, about 30% of opioid-dependent patients experienced prolonged hospitalization compared to 22% of non-dependent patients, with a relative risk of 1.36 (99.5% CI, 1.20–1.53).

Other postoperative complications were observed at generally comparable rates between opioid-dependent and non-dependent patients. These included surgical site infections (0.3% vs. 0.1%), urinary tract infections (2.7% vs. 2.5%), dural tears (1.2% vs. 0.6%), acute renal failure (2.9% vs. 2.7%), cardiac complications (5.8% vs. 4.7%), and stroke (0.2% vs. 0.1%). Although slight differences were noted, none represented large disparities. Importantly, in-hospital mortality remained exceptionally low in both groups, with no statistically significant difference (0.06% in opioid-dependent vs. 0.19% in non-dependent patients), highlighting the overall safety of the surgical setting despite the presence of opioid dependence.

### Results of Propensity Score Weight (PSW)

After applying propensity score weighting (PSW) to account for baseline differences between groups, several postoperative complications remained significantly more prevalent among opioid-dependent patients, as outlined in Table 4. Blood loss anemia emerged as the most notable complication, with opioid dependence associated with 79% increased odds (OR 1.79, 99.5% CI 1.53–2.11, *p* < 0.005). Respiratory complications were also significantly elevated (OR 2.17, 99.5% CI 1.54–3.06, *p* < 0.005), as were surgical site infections (OR 3.94, 99.5% CI 1.26–12.2, *p* = 0.001) and cardiac complications (OR 1.53, 99.5% CI 1.15–2.03, *p* < 0.005).

In addition, 35% of opioid-dependent patients incurred high hospital charges versus approximately 30% of their non-dependent counterparts, with a relative risk of 1.17 (99.5% CI, 1.12–1.22; *p* < 0.005). Specifically, 30% of opioid-dependent patients experienced prolonged hospital stays compared to approximately 25% of non-dependent patients, with a relative risk of 1.20 (99.5% CI, 1.15–1.26; *p* < 0.005). These findings underscore a persistent increase in healthcare burden attributable to opioid dependence, even after adjusting for confounding factors.

**Figure 1:**
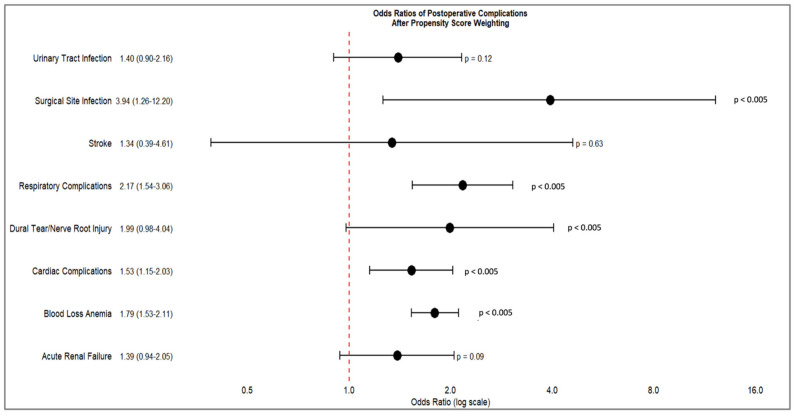
Forest plot of odds ratio of postoperative complications after propensity score weighting.

Other complications, such as urinary tract infections (OR 1.40, *p* = 0.12), acute renal failure (OR 1.39, *p* = 0.09), dural tear or nerve root injury (OR 1.99, *p* = 0.054), and stroke (OR 1.34, *p* = 0.63), did not reach statistical significance after adjustment, although some trended toward increased risk. These findings, summarized in Table 5 and Figure 1, indicate that while PSW helped reduce confounding, important and clinically meaningful disparities in postoperative outcomes persist between opioid-dependent and non-dependent patients.

## 4. Discussion

In this large, national cohort study of 597,455 patients undergoing spinal surgery, opioid dependence was associated with distinct demographic, clinical, and perioperative characteristics that influenced postoperative outcomes. Opioid-dependent patients were significantly younger and more likely to be treated in large, urban teaching hospitals, with a notable geographic concentration in the West and Midwest regions. These patients exhibited a distinct comorbidity profile, with higher rates of alcohol abuse, chronic obstructive pulmonary disease (COPD), sleep apnea, chronic anemia, and chronic kidney disease, while traditional cardiovascular risk factors such as hypertension and diabetes were comparably prevalent between groups. Propensity-adjusted analyses confirmed that opioid dependence was an independent risk factor for increased postoperative complications, particularly blood loss anemia, respiratory complications, surgical site infections, and cardiac events. Moreover, opioid-dependent patients demonstrated prolonged hospital stays and higher healthcare costs, reinforcing the economic burden associated with opioid-related morbidity in surgical populations. While some complications, such as urinary tract infections and acute renal failure, showed attenuated differences after adjustment, the overall trend suggests that opioid dependence is a significant determinant of adverse perioperative outcomes.

Opioid dependence, recognized as a public health epidemic, has been the subject of extensive research and institutional guidelines [13,15,16,17,18]. Its impact on surgical outcomes has been examined across multiple disciplines [9,10,14,19]. While prior studies have suggested a correlation between opioid dependence and adverse surgical outcomes, many were limited by small cohort sizes or have become outdated [9,10,12,20]. One study reported an increasing prevalence of opioid dependence among lumbar fusion patients, identifying higher postoperative complication rates and prolonged hospital stays [21]. It utilized the National Inpatient Sample (NIS) database from 2003 to 2014, relying on the International Classification of Diseases, Ninth Revision, and Clinical Modification (ICD-9-CM). In contrast, our study leverages the Tenth Revision (ICD-10-CM), which provides more precise and comprehensive coding for diagnoses and procedures, allowing for a more accurate assessment of contemporary trends in opioid dependence among lumbar fusion patients.

Over the past few years, we have seen big shifts in opioid use. Studies of opioid prescriptions from 2016 to 2021 show that doctors are prescribing them less often, and fewer people are using them long-term [22,23]. But here is something interesting: another study found that non-fatal overdoses actually increased during this same period, which tells us that opioid use is a tricky issue [24]. So, what does this mean for people who are having spinal fusion surgery and are dependent on opioids? We need to conduct more research to see if these new prescribing habits are actually helping them have better outcomes after their surgery.

Our study identified a significantly higher incidence of blood loss anemia among opioid-dependent patients (OR 1.79, *p* < 0.001). We initially hypothesized that opioids may interfere with the clotting cascade, potentially contributing to increased perioperative blood loss. However, our findings did not support this hypothesis. Conversely, existing literature suggests that opioid use may impair antiplatelet medication absorption, which could exacerbate perioperative hemostatic challenges [25,26,27]. These findings underscore the need for further research into the interplay between opioid dependence and coagulation mechanisms in surgical patients.

From a clinical perspective, our findings emphasize the critical importance of preoperative screening for opioid dependence and the potential perioperative complications associated with chronic opioid use. The growing body of evidence supporting non-opioid-based pain management protocols demonstrates promising results in postoperative pain control [28,29,30]. Therefore, multimodal analgesic strategies should be considered to mitigate the adverse impact of opioid dependence on surgical outcomes and facilitate enhanced recovery following lumbar spine fusion.

This study comes with certain limitations due to the use of the National Inpatient Sample (NIS) database, which may affect its applicability in orthopedic surgery research. First, coding inaccuracies remain a persistent challenge, as administrative datasets rely on procedural and diagnostic codes rather than direct clinical validation. Such discrepancies may introduce misclassification bias, potentially affecting the reliability of reported outcomes. The second limitation of our study is the spectrum of opioid dependence, which varies based on the duration and intensity of opioid consumption. Additionally, we are unable to determine whether a patient previously underwent rehabilitation or successful opioid cessation but still retains an opioid dependence diagnostic code in their medical records. This limitation may lead to potential misclassification and affect the accuracy of our analysis regarding the true burden of opioid dependence in patients undergoing lumbar spine fusion. Third, the NIS database exclusively captures inpatient encounters, thereby precluding the assessment of outpatient perioperative factors that could significantly influence surgical outcomes. This limitation may result in an underestimation of postoperative complications that manifest beyond the index hospitalization or are managed in ambulatory settings. Fourth, the absence of longitudinal follow-up restricts our ability to evaluate critical postoperative endpoints, such as implant longevity, late-onset infections, or delayed mechanical failures. Moreover, this study reports hospital charges rather than estimated costs, which may limit direct financial interpretation. Additionally, dollar amounts were not adjusted for inflation across the study period. These choices were made to maintain consistency with prior studies and due to the primary focus on relative group comparisons rather than temporal economic trends. Consequently, conclusions regarding long-term LF outcomes, particularly among opioid-dependent patients, remain incomplete. Future research integrating linked databases or prospective cohort designs is warranted to overcome these limitations and provide a more comprehensive understanding of the factors influencing long-term surgical success.

This study offers several important strengths worth highlighting. To the best of our knowledge, it is the first to conduct a comprehensive analysis using multiple years of data coded with the ICD-10 system—making our findings highly relevant to current clinical practice and modern reimbursement structures. The use of standardized coding not only ensures consistency but also allows for meaningful comparisons with future studies and supports more accurate epidemiological insights. Additionally, by including data spanning six years, our analysis benefits from increased statistical power and a more representative sample than many previous investigations. This extended dataset enables us to better understand evolving trends in hospitalization costs and length of stay, offering valuable perspectives on the economic and institutional factors shaping lumbar fusion outcomes. Ultimately, these insights can help guide smarter resource use, improve perioperative care, and support more informed health policy decisions.

## 5. Conclusions

In conclusion, opioid dependence remains a complicating factor in postoperative outcomes of LF, imposing additional burdens on healthcare systems through prolonged hospitalizations and increased costs. Future studies are warranted to investigate the impact of opioids on coagulation mechanisms, as opioid use may influence thrombotic or bleeding tendencies, potentially affecting postoperative outcomes. Additionally, further research should explore the spectrum of opioid dependence and its varying degrees of impact on surgical complications, recovery trajectories, and long-term functional outcomes. Understanding these relationships could lead to optimized perioperative pain management strategies and improve risk stratification for opioid-dependent patients undergoing lumbar spine fusion.

## Figures and Tables

**Table 1:** ICD-10 and procedure codes used for case selection and variable definition.

Category	Variable	Codes
**Lumbar Fusion Procedures**	Lumbar Fusion	0SG0070, 0SG0071, 0G007J
		0SG00A0, 0SG00AJ, 0SG00J0
		0SG00J1, 0SG00JJ, 0SG00K0
		0SG00K1, 0SG0370, 0SG0371
		0SG037J, 0SG03A0, 0SG03AJ
		0SG03J0, 0SG03J1, 0SG03K0
		0SG03K1, 0SG03KJ, 0SG0471
**Comorbidities**	Opioid Dependence	F11.2x
	Type 2 Diabetes	E11.x
	Hypertension	I10
	Dyslipidemia	E78.x
	Sleep Apnea	G47.3x
	Anemia	D64.x
	Alcohol Abuse	F10.x
	Smoking	Z72.0
	Chronic Kidney Disease	N18.x
	COPD	J44.x
**Complications**	Surgical Site Infection	T81.4x
	Urinary Tract Infection	N39.x
	Respiratory Complications	J96.x, J95.x, J80.x, J15.x, J18.x
	Dural Tear/CSF Leak/Nerve Root Injury	G96.11, G96.00, G96.02, S34.x
	Acute Renal Failure	N17.x
	Embolism (PE, DVT)	I26.x, I82.4x
	Blood Loss	D62.x
	Cardiac Complications	I44.x, I20.x, I49.x, I50.x, I51.x, I23.x, R96.x, I21.x, I22.x, I24.x, I46.x, T81.1
	Stroke	I63.x, G45.x

**Table 2:** Clinical characteristics and hospital charges by opioid use.

Characteristic	Not Opioid Dependent *n* = 589,740	Opioid Dependent *n* = 7715	*p*-Value
Demographics			
Age, mean (SD)	62.16 (12.09)	58.23 (11.36)	<0.001
Female, n (%)	331,750 (56.3)	4450 (57.7)	0.25
Hospital Bed Size, n (%)			
Small	162,105 (27.5)	1165 (15.1)	<0.001
Medium	149,380 (25.3)	1645 (21.3)	<0.001
Large	278,255 (47.2)	4905 (63.6)	<0.001
Hospital Location/Teaching, n (%)			
Rural	23,100 (3.9)	305 (4.0)	<0.001
Urban Non-teaching	133,695 (22.7)	1220 (15.8)	<0.001
Urban Teaching	432,945 (73.4)	6190 (80.2)	<0.001
Hospital Region, n (%)			
Northeast	95,440 (16.2)	880 (11.4)	<0.001
Midwest	140,800 (23.9)	2110 (27.3)	<0.001
South	242,980 (41.2)	2445 (31.7)	<0.001
West	110,520 (18.7)	2280 (29.6)	<0.001
Race, n (%)			
White	471,060 (79.9)	6225 (80.7)	0.008
Black	47,125 (8.0)	650 (8.4)	0.008
Hispanic	31,300 (5.3)	415 (5.4)	0.008
Asian	7295 (1.2)	15 (0.2)	0.008
Other	32,960 (5.6)	410 (5.3)	0.008
Primary Payer, n (%)			
Medicare	288,315 (48.9)	3885 (50.4)	<0.001
Medicaid	34,490 (5.8)	990 (12.8)	<0.001
Private	223,510 (37.9)	2275 (29.5)	<0.001
Other	43,425 (7.4)	565 (7.3)	<0.001
Comorbidities, n (%)			
Type 2 Diabetes	129,170 (21.9)	1715 (22.2)	0.75
Hypertension	314,400 (53.3)	4045 (52.4)	0.49
Dyslipidemia	247,720 (42.0)	3030 (39.3)	0.02
Sleep Apnea	95,790 (16.2)	1455 (18.9)	0.005
Chronic Anemia	27,720 (4.7)	475 (6.2)	0.01
Alcohol Abuse	6085 (1.0)	265 (3.4)	<0.001
Smoker	4220 (0.7)	60 (0.8)	0.77
Chronic Kidney Disease	36,770 (6.2)	595 (7.7)	0.02
COPD	43,690 (7.4)	985 (12.8)	<0.001

**Table 3:** Baseline characteristics after propensity score weighting.

Characteristic	Overall	Not Opioid Dependent *n* = 7715	Opioid Dependent *n* = 7715	*p*-Value
Age, mean (SD)	61.88 (11.83)	62.11 (12.08)	61.62 (11.54)	0.20
Female (%)	55.7	56.3	55.0	0.45
DM type 2 (%)	22.2	21.9	22.5	0.65
Hypertension (%)	53.3	53.3	53.3	0.98
Dyslipidemia (%)	42.7	42.0	43.5	0.33
Sleep apnea (%)	16.2	16.3	16.2	0.92
Anemia (%)	4.6	4.7	4.4	0.66
Alcohol abuse (%)	1.1	1.1	1.2	0.60
Smoking (%)	0.7	0.7	0.7	0.93
Renal disease (%)	6.2	6.3	6.1	0.85
Lung disease (%)	7.8	7.5	8.1	0.40
Hospital Size (%)				
Small	27.2	27.3	27.0	0.88
Medium	24.9	25.3	24.5	
Large	47.9	47.4	48.5	
Hospital Type (%)				
Rural	4.3	3.9	4.7	0.71
Urban non-teaching	22.2	22.6	21.8	
Urban teaching	73.5	73.5	73.5	
Region (%)				
Northeast	15.8	16.1	15.4	0.62
Midwest	23.0	23.9	21.9	
South	41.6	41.1	42.2	
West	19.6	18.9	20.5	
Insurance (%)				
Medicare	48.2	48.9	47.4	0.73
Medicaid	6.0	5.9	6.1	
Private	38.2	37.8	38.6	
Other	7.6	7.4	7.9	

**Table 4:** Postoperative complications and hospital outcomes by opioid use status after PSW.

Complications	(%)	Effect	Estimate	Lower CI	Upper CI	*p*-Value
Urinary Tract Infection	2.6	OR	1.40	0.90	2.16	0.12
Stroke	17.8	OR	1.34	0.39	4.61	0.63
Surgical Site Infection	0.2	OR	3.94	1.26	12.2	<0.005
Blood Loss Anemia	17.8	OR	1.79	1.53	2.11	<0.005
Cardiac Complications	5.3	OR	1.53	1.15	2.03	<0.005
Acute Renal Failure	2.8	OR	1.39	0.94	2.05	0.09
Dural Tear/Nerve Root Injury	0.9	OR	1.99	0.98	4.04	0.054
Respiratory Complications	0.3	OR	2.17	1.54	3.06	<0.005
**Hospital Outcomes**						
Prolonged LOS (75th %) ($)		RR	1.17	1.12	1.22	<0.005
Length of stay		RR	1.20	1.15	1.26	<0.005

ES, estimate; OR, odds ratio; RR, relative risk. Note: All confidence intervals are 99.5% to account for Bonferroni correction.

**Table 5:** Postoperative complications and hospital outcomes by opioid use status before PSW.

Complications	ES	Estimate	Lower CI	Upper CI	*p*-Value
Stroke	OR	1.51	0.48	4.74	0.48
Blood Loss Anemia	OR	1.84	1.62	2.08	<0.005
Cardiac Complications	OR	1.32	1.07	1.64	0.01
Acute Renal Failure	OR	1.36	1.00	1.84	0.04
Dural Tear/Nerve Root Injury	OR	2.21	1.40	3.49	<0.005
Respiratory Complications	OR	2.10	1.61	2.73	<0.005
Urinary Tract Infection	OR	1.49	1.09	2.03	0.01
Surgical Site Infection	OR	3.27	1.34	8.02	0.009
**Hospital Outcomes**					
Prolonged LOS (75th %) (USD)	RR	1.25	1.10	1.42	<0.005
Length of stay	RR	1.36	1.20	1.53	<0.005

ES, estimate; OR, odds ratio; RR, relative risk. Note: All confidence intervals are 99.5% to account for Bonferroni correction.

## Data Availability

Restrictions apply to the availability of these data. Data were obtained from HCUP and are available [https://hcup-us.ahrq.gov/] with the permission of HCUP.

## Abbreviations

HCUP	Healthcare Cost and Utilization Project
ICD-10	International Classification of Diseases, 10th Revision
LF	Lumbar Fusion
LOS	Length of Stay
NIS	Nationwide Inpatient Sample
SPSS	Statistical Package for the Social Sciences
